# 921. Preliminary Survey Analysis of Adult Adaptive Sport Burn Survivor Retreats; a Blueprint for Resilience/Recovery

**DOI:** 10.1093/jbcr/irag033.446

**Published:** 2026-04-06

**Authors:** Roselle E Crombie, Cindy Rutter, Josh Mishell, Daniel W Chacon, Phil Fidler, Rebekah R Allely

**Affiliations:** Connecticut Burn Center/Yale New Haven Health, Westport, CT; 1 Amazing Life, Carlsbad, CA; REACH Burn Foundation, Denver, CO; Phoenix Society for Burn Survivors, Fresno, CA; Texas Tech El Paso, El Paso, TX; Medstar Washington Hospital Center, Reston, VA

## Abstract

**Introduction:**

As survivors increase with improvements in burn care and prevention, many are ill equipped to face their future. Adult burn survivors describe long-term psychosocial challenges, difficulty with vocations and limited access to therapy. In contrast to support for pediatric burns, very few programs address the gap in programming for adults. A retreat program was developed to address both the psychosocial needs and physical challenges faced with the goal of empowerment. Woven in were activities of adaptive sports, art therapy, inspirational talks, and shared life skills. The program was then surveyed to evaluate impact and survivor resilience.

**Methods:**

Three surveys were designed modeling with permission the RISC-10, a validated resilience scale. Alongside this, a new set of Behavioral Resilience Indicators (BRIs) to capture specific, actionable behaviors that support resilience growth were developed.

Pre-Retreat – Demographic questions + RISC-10 (baseline).

Post-Retreat – RISC-10 + *optional (BRI Intentions) + Participant Satisfaction Survey.

6-Month Follow-Up – RISC-10 + BRI Actions + brief reflection check-in.

Post retreat virtual interviews were done around 90 days. Descriptive statistics were applied but plans for multivariate analysis to control for age, ethnicity, years from burn to understand the effect of the retreat are ongoing. Three cohorts included a total of 30 culturally diverse adult burn survivors, aged 26–62, > one year post-injury (range: 1–40 years). Two cohorts participated in a fall retreat over three- four days with summer-based activities and attended trauma-informed workshops focused on emotional processing and narrative identity reconstruction. The third cohort participated in a three day winter adaptive snow sports program retreat and similar workshops.

**Results:**

All three cohorts demonstrated significant post retreat improvement in self perception, overall mental health and profound impact of community and peer support. Excerpts include powerful indicators with 100% of participants affirming a yes response include; “In the past 6 mos were you able to break down challenges into smaller manageable steps?’, “Did you accomplish a personal or professional goal?’. Change was also evident when comparing the pre and post questions in terms of emotional self assessment.

**Conclusions:**

Although this is a preliminary assessment of the impact of an adult based retreat, positive changes in participants are evident at the interval of 6-12 months. Other survey questions describing things that have changed since the retreat include, usage of acquired skills to deal with conflict, acquisition of jobs, taking on new challenges and starting families. Ongoing study of the impact of this adult programming is key to providing lessons of resilience.

**Applicability of Research to Practice:**

This analysis is meant to study a unique but impactful retreat and it's effects on psychosocial well being for survivors.

**Funding for the study:**

N/A.

